# Evaluation of Subclinical Retinal Disease in Patients Affected by Systemic Lupus Erythematosus with No Evidence of Ocular Involvement—An Optical Coherence Tomography Angiography Original Study

**DOI:** 10.3390/jcm11247417

**Published:** 2022-12-14

**Authors:** Małgorzata Mimier-Janczak, Dorota Kaczmarek, Krzysztof Proc, Marta Misiuk-Hojło, Radosław Kaczmarek

**Affiliations:** 1 Department and Clinic of Ophthalmology, Wroclaw Medical University, 50-556 Wroclaw, Poland; 2 Ophthalmology Clinical Centre Spektrum, 53-334 Wroclaw, Poland; 3 Department of Rheumatology and Internal Medicine, Wroclaw Medical University, 50-556 Wroclaw, Poland

**Keywords:** angiography (OCTA), systemic lupus erythematosus, retinal capillaries, subclinical retinal changes, vessel density

## Abstract

Lupus retinopathy is the second most common eye involvement in systemic lupus erythematosus (SLE), associated with significant visual deterioration and well-known negative prognostic factor for survival. Ocular manifestation in SLE, relating the retina, ranges from asymptomatic vascular involvement to vision devastating vascular occlusions. Subclinical microvascular changes are undetectable in slit lamp examination, hence are underdiagnosed. Optical coherence tomography angiography (OCTA) is a novel, easy to interpret and non-invasive technique that allows retinal vessels visualization. OCTA simplifies clinical approach and measures the severity of decreased perfusion. The aim of the study was to demonstrate the retinal vascularization in a subclinical stage of ocular involvement in a cohort of SLE patients. Thirty-three patients (57 eyes) diagnosed with SLE were enrolled into the study group and 31 healthy individuals (56 eyes) into the control group. Vessel density reduction in parafovea, inferior and nasal quadrants of superficial retinal capillary plexus in a cohort of SLE patients was found. Among study group kidney involvement was associated with further microvasculature reduction. Knowing that retinal involvement may precede other organs impairment, early detection of retinal impairment and use of OCTA as a screening modality, may decrease overall disease morbidity.

## 1. Introduction

Systemic lupus erythematosus (SLE) is a heterogeneous connective tissue disease primarily affecting young women. Clinical presentation of this autoimmune disease may vary from mild cutaneous involvement to severe cardiovascular life-threatening complications.

Lupus retinopathy is the second most common eye involvement in SLE, associated with significant visual deterioration and well-known negative prognostic factor for survival. Retinopathy may be secondary to insufficient disease control. It may have different clinical manifestations such as microangiopathy, vascular-occlusive changes or true vasculitis [1]. Immune complex-mediated microangiopathy is the most common manifestation, affecting from 3 to 29% patients, with typically mild course and good visual outcome, unless the macula is involved. It is caused by inflammatory reaction secondary to adhesion of immune complexes deposits to the basement membrane of endothelium [2,3,4]. In fundus it is characterized by cotton wool spots, small intraretinal hemorrhages, microaneurysms, hard exudates or retinal edema, as well as papilledema [1,2,3,5]. Retinal vascular occlusions are located mainly in arteries, or in veins. Lack of perfusion in retinal capillaries that results in ischemia, may lead to neovascularization, hemorrhage in the vitreous chamber, retinal detachment, or neovascular glaucoma. Severe vaso-occlusive disease, such as central retinal artery occlusion and central retinal vein occlusion, is observed in less than 1% of patients but is related to significant visual loss [2]. It is associated with fibrinoid degeneration or necrosis and is more prevalent in patients with antiphospholipid syndrome and antiphospholipid antibodies [6]. True retinal vasculitis, inflammation of venules or arterioles, is uncommon with acute presentation [1,7].

Lupus choroidopathy is less common than retinopathy, but it is also an indicator of high disease activity [8]. Its manifestations include serous retinal detachment, detachment of retinal pigment epithelium or retinal pigment epitheliopathy [5,8]. Drusen-like-deposits are more frequent in SLE patients [9,10]. They were present regardless of kidney involvement, but patients without nephritis have smaller and less numerous drusen [10].

Optical coherence tomography angiography (OCTA) is safer, faster, and easier to perform than fluorescein angiography and is used for the same medical indications [11]. It is a novel, non-invasive technique that allows retinal vessels visualization, in form of a high-resolution angiographic maps. OCTA simplifies clinical approach and measures the severity of decreased perfusion. Forte et al. confirmed OCTA strength in the detection of early microangiopathy in the absence of changes in visual acuity [12].

Retinal manifestation in SLE ranges from asymptomatic vascular involvement to vision devastating vascular occlusions. Subclinical microvascular changes are undetectable in slit lamp, hence are underdiagnosed. Several authors have showed an impaired retinal vessel density (VD) and reduced foveal avascular zone (FAZ), measured by OCTA, in patients affected by SLE with no ocular symptoms and with no history of ocular disease [12,13,14,15,16,17,18]. In these papers, there were shown conflicting results regarding the effect of SLE on FAZ area, effect of nephritis on VD and correlation between the disease activity indices and OCTA parameters [1]. Considering promising papers, our aim was to confirm abovementioned results and correlate them with extended rheumatological data. Our hypothesis was that OCTA would show altered microcirculation in ophthalmologically asymptomatic SLE patients and that level of impairment is correlated with disease activity.

Primary objective was to describe the retinal vascularization, by means of VD and FAZ, in a subclinical stage of ocular involvement and secondary objective was to investigate association between the morphology of the retina, SLE activity and damage index, kidney and CNS involvement, hydroxychloroquine (HCQ) and chloroquine (CQ) cumulative doses.

## 2. Materials and Methods

The study was approved by the Bioethics Committee of the Wroclaw Medical University (Poland) and before the examination all participants provided written informed consent.

Thirty-three patients (57 eyes) diagnosed with SLE, according to the American College of Rheumatology classification criteria, were enrolled into the study group and 31 healthy individuals (56 eyes) into the control group. Because of the poor quality of the scans, 3 patients (4 eyes) were excluded from the study group. Exclusion criteria included: age over 75 and below 18 years, any signs or history of SLE retinopathy, other eye diseases, drug-induced retinal damage, refractive error greater than –5 diopters, history of trauma or history of eye surgery within last 6 months. Patients from study group were asymptomatic, with no evidence of ocular involvement on fundus examination and in OCT. The study lasted from July 2019 to October 2021. Each patient enrolled into the study, was provided with a full ophthalmological examination, including best corrected visual acuity (BCVA), air puff intraocular pressure measurement (IOP), examination of the anterior and posterior segment of the eye in a slit lamp after pharmacological pupil dilatation with 1% Tropicamide, and OCTA examination. The results of laboratory and diagnostic tests, as well as questionnaires regarding SLE, were analyzed by experienced rheumatologist to quantify disease damage. The Systemic Lupus Erythematosus Disease Activity Index 2000 (SLEDAI-2K) and The Systemic Lupus International Collaborating Clinics/American College of Rheumatology Damage index (SLICC/ACR DI) were used to assess the disease severity and activity.

Regarding the numerical values of some parameters—concentration of: anti dsDNA [IU/mL] < 10 were considered as 0, >800 as 801, ACL IgM, IgG [U/mL] and anti-β2 GPI IgM, IgG [RU/mL] < 2 were considered as negative, ≥2 as positive, C4 [g/L] < 0.08 were considered as negative, ≥0.08 as positive.

All the patients underwent detailed retinal analysis with an automatically centered on the fovea, 6 × 6 mm OCTA scan and a radial OCT B-scan, using Swept-Source Optical Coherence Tomography Angio DRI OCT Triton Plus (Topcon Corp., Tokyo, Japan, 2015). OCTA evaluation included VD measurement in superficial retinal capillary plexus (SRCP) and deep retinal capillary plexus (DRCP) in five regions, similar to the Early Treatment Diabetic Retinopathy Study (ETDRS) subfields: fovea, superior, inferior, nasal, temporal and then manually averaged by authors into parafoveal and whole en face vessel density. The built-in software and algorithm were used for automated layer segmentation and vessel density measurement. SRCP was defined from 2.6 μm below internal limiting membrane (ILM) to 15.6 μm below the inner plexiform (IPL) and inner nuclear layers (INL) (IPL/INL) and DRCP 15.6 μm to 70.2 μm below IPL/INL.

To determine FAZ parameters from the SRCP: area, perimeter and circularity index, the OCTA images were exported as 320 × 320 pixels image and manually outlined and calculated using Adobe Photoshop version 23.0.1 (Adobe, Inc., San Jose, CA, USA, 2021) by the same, masked, experienced ophthalmologist. Circularity index was defined by 4π × area/perimeter^2^. Value equal to 1.0 indicates a perfect circle, while values below 1.0 less circular shape of FAZ.

Data from both eyes of each patient was averaged prior to statistical analysis. Authors decided to use averaged data, because of systemic character of the SLE that should evenly affect both eyes. The averaged values, in authors opinion, demonstrates the patient’s condition more clearly. They also lower the impact of an atypical patient course—significant asymmetrical eye involvement—on the final results.

Statistical analysis was performed using a STATISTICA 13.3 (StatSoft, Inc., Tulsa, OK, USA, 2017). The results with *p* < 0.05 were considered statistically significant. The Kolmogorov-Smirnow test was used to assess the normality of the distribution of the investigated quantitative features. Statistical analysis examining the differences between the individual measured parameters in the groups was performed using the U-Mann-Whitney test. The correlation between the measured parameters and quantitative clinical data was performed using the Spearman’s rank correlation test. ANOVA (analysis of variance) test was used for more than two groups comparison. We have performed multiple regression analysis between statistically significant differences between control and study group with a check for autocorrelation using Durbin-Watson test and have added this value to the footnote of each table (Supplementary Tables S1–S5). We have done power analysis for our main comparison (OCTA parameters between SLE patients and control group)—Supplementary Table S6, and for multiple regression analysis—Supplementary Table S7.

## 3. Results

All the collected data regarding patients’ characteristics, SLE activity and treatment are summarized in Table 1 and Table 2.

There was no significant difference in age, gender, IOP, vessel density in DRCP and FAZ perimeter between SLE patients and healthy controls. There was no difference among analyzed parameters between men (*n*—5) and women (*n*—25) in study group, as well as, in whole cohort of patients (*n*—52, *n*—9).

Visual acuity was significantly lower (*p* = 0.019) in SLE patients than in the control group. The eyes from SLE patients had lower superficial parafoveal vessel density (*p* = 0.018) as well as in inferior (*p* = 0.038) and nasal (*p* = 0.018) quadrants compared to the control group. FAZ area and FAZ circularity index (*p* = 0.035, *p* = 0.008) were reduced in patients suffering from SLE. Comparison of OCTA parameters between study and control group is shown in Table 3.

Statistically significant positive correlation between HCQ cumulative dose and vessel density in SRCP (R = 0.422, *p* = 0.020) and DRCP (R = 0.382, *p* = 0.037) in superior quadrants was demonstrated. VD in superior quadrant of SRCP was also increased in SLE patients treated with HCQ for more than 5 years compared to patients with HCQ therapy for less than 5 years. (*p* = 0.022). Comparison of OCTA parameters among SLE patients based on HCQ treatment duration is shown in Table 4.

Twelve patients with SLE were taking chloroquine. There was lower VD in temporal quadrant of DRCP compared to patients without chloroquine therapy, but no difference between chloroquine cumulative dose and OCTA parameters, including VD. Eleven patients from study group were using azathioprine. There was no difference in analyzed parameters compared to SLE patients that were not using azathioprine. Thirteen patients from study group were using methotrexate. Our study revealed lower VD in superficial capillary plexus in inferior quadrant in SLE patients treated with methotrexate (*p* = 0.03). Comparisons of OCTA parameters among SLE patient using methotrexate, azathioprine and chloroquine are summarized in Table 5, Table 6 and Table 7.

Lupus nephropathy was observed in 6 patients in our study group. Patients with SLE and nephritis had lower whole en face superficial vessel density (*p* = 0.004), superficial parafoveal density (*p* = 0.007) and superficial density in nasal (*p* = 0.02) and temporal (*p* = 0.02) quadrants compared with patients with SLE without kidney involvement. Comparison of OCTA parameters among SLE patient with kidney involvement is presented in Table 8.

Among study group a parafoveal VD in DRCP decreased with age.

No correlations were revealed between examined OCTA parameters and C4, LAC, AnuA, anty-ß2 GPI IgM and IgG, Anti dsDNA and ACL IgG concentrations and ESR. Association between increase in ACL IgM concentration on decrease of VD in DRCP in fovea and increase in C3 on decrease VD in DRCP in superior quadrant was observed—but these results may be incidental because of small study group.

The effect of neuropsychiatric SLE, rituximab, leflunomide, cyclophosphamide and prednisolone usage on the retinal changes was also analyzed. Due to the insufficient size of these groups (neuropsychiatric SLE—3, rituximab—1, leflunomide—1, cyclophosphamide—4, prednisolone—3), the results are unreliable.

No correlation was found between the SLICC/ACR and the SLEDAI-2K scores and the retinal vessel density in superficial and deep retinal capillary plexus and FAZ.

We have performed multiple regression analysis between statistically significant differences between control and study group (VD SRCP inferior, VD SRCP nasal, VD SRCP parafovea, FAZ area and FAZ circularity index) with the following variables: age, gender, visual acuity, disease duration, disease severity, renal involvement, HCQ total dose, chloroquine use, azathioprine use, methotrexate use. The analysis showed that in the case of VD SRCP parafovea, only kidney involvement is a statistically significant predictor and almost reach statistical significance for VD SRCP nasal (*p* = 0.052773). For the FAZ area, such a predictor is the use of azathioprine. The FAZ circularity index does not have such a predictor among the listed data (Supplementary Tables S1–S5).

Values in Table 1, Table 2, Table 3, Table 4, Table 5, Table 6, Table 7 and Table 8 are presented as mean ± standard deviation.

## 4. Discussion

The microvascular changes in SLE play an extremely important role in disease morbidity and mortality. The involvement of posterior segment of the eye may mirror other organs vascular involvement [19,20,21]. Well known clinical tools that can assess the morphological changes in patients’ microvasculature are capillaroscopy, laser Doppler flowmetry and laser speckle contrast imaging [21]. The eye, through the pupil, allows for a view of small vessels and capillaries of the retina and choroid.

Our study revealed lower VD in parafovea, inferior and nasal quadrants of SRCP and reduction of FAZ area and FAZ circularity index in SLE patients. Our results concerning FAZ are at odds to Forte et al., Pichi et al., Mihailovic et al. and An et al. findings [12,14,15,22]. Some authors link enlargement of the FAZ with hypoperfusion in the retinal vessels, tissue hypoxia, and retinal cell death in patient with subclinical SLE [15]. However, large individual variations of the FAZ size and shape in healthy subject have been found [23]. Also, the measurement of the FAZ area may be challenging as the area is very irregular with many entering capillaries.

Bao et al. found out significant decrease in vascular density in the SRCP among patients diagnosed with lupus but without signs of lupus retinopathy and decrease in VD in both SRCP and DRCP in patients with lupus retinopathy [17]. According to Bao et al. subclinical retinal microvasculature impairment precedes proper lupus retinopathy development, making it the early marker of lupus retinopathy. The process may be caused by immune-complex deposition in vessels endothelium leading to retinal nerve fiber layer (RNFL) and ganglion cell layer (GCL) atrophy [15,17,24]. Authors speculate that SRCP and then DRCP impairment led to insufficient oxygen and nutrient supply to inner retina causing changes in the retina structure contributing to the development of lupus retinopathy. On the other hand, Arfeen et al. results showed the decrease VD in the DRCP in all sectors, while some of the quadrants in the SRCP showed no significant difference [16]. An et al. achieved similar results [22]. Authors suggest that DRCP is the most vulnerable to early impairment, because of its anatomical position and the role of inner retina blood supply, compared to other vessel networks. DRCP impairment can be consider as an early disease activity and damage biomarker. DRCP tends to gradual obstruction follows by hemodynamic dysfunction [16,25]. As a consequence, it may lead to photoreceptors integrity loss, but future studies are necessary to confirm the thesis [26].

Visual acuity was also statistically significant lower in SLE patients, which may be secondary to the degree of capillary loss, but this correlation is uncertain, and data is limited [27,28,29]. Some authors associate decreased visual acuity with the perfusion status of DRCP [28,30]. All enrolled patients were clinically asymptomatic—they did not report any impairment in visual acuity. Decreased BCVA (logMAR) in ophthalmological examination, was found only in five eyes—four eyes 0.1, one eye 0.2 in study group. This clinically insignificant difference happened to be statistically significant.

In contrast to Conigliaro et al. and Arfeen et al. results., our study has revealed no correlation between the SLICC/ACR and the SLEDAI-2K scores and the retinal microvascular alterations [13,16]. Our results were compatible with Pichi et al. [15]. However, the association between retinal vessels remodeling with the involvement of other organs was shown. Patients with SLE and nephritis had lower whole en face superficial vessel density, superficial parafoveal density and superficial density in nasal and temporal quadrants compared with patients with SLE without kidney involvement. In Conigliaro et al. study patients with kidney involvement had also reduced superficial parafoveal vessel density compared with SLE patients without nephritis [13].

SLE patients are especially vulnerable group for retinal ischemia development. Both, SLE itself and SLE drug therapy (hydroxychloroquine, chloroquine, glucocorticosteroids, methotrexate, cyclosporine-A), can irreversibly damage the retina and be responsible for severe visual deterioration, as well as may overlap and confuse the study findings [31,32,33]. HCQ is widely used in SLE treatment with good efficacy, disease control, patient’s tolerance but few side effects. Toxic drug-related retinopathy is one of the most dangerous side effects associated with HCQ cumulative dosage [34]. After 5 years of HCQ therapy, the risk of toxic retinopathy increases dramatically, while up to 5 years of treatment is less than 1% [35]. In the current study VD in superior quadrant of SRCP was increased in SLE patients treated with HCQ for more than 5 years compared to patients with HCQ therapy for less than 5 years. Our study revealed also positive correlation between HCQ cumulative dose and vessel density in SRCP and DRCP. These positive correlations might be indicative of protective function of HCQ on ocular microvasculature, even if it relates to longer SLE duration. Our study revealed lower VD in SRCP in inferior quadrant in SLE patients treated with methotrexate compared to SLE patients with no history of methotrexate therapy. Methotrexate may be responsible for ischemic retinal complications but futured studies are necessary to evaluate the correlation with VD [18].

Current paper found a negative correlation, among study group, between age and parafoveal VD in DRCP. Those findings are in alignment with Conigliaro et al. results where the correlation between age and superficial whole en face density, superficial foveal density, superficial parafoveal density and deep whole en face density was also showed [13]. VD tends to decline during process of aging [36,37]. Because vessel density correlates with age and may alternate the results obtained in studies detecting retinal microvascular changes in long-lasting systemic disease, this impact should be considered in futures studies.

Altered microcirculation in retina has been found in many autoimmune diseases, such as Crohn’s disease, ulcerative colitis, and rheumatoid arthritis [38]. Debourdeau et al. have shown reduced VD in SRCP and radial peripapillary capillaries and larger FAZ in patients with severe Crohn’s disease [38]. These patients had increased FAZ area and reduced VD in radial peripapillary capillaries compared to patients with moderate Crohn’s disease. The study suggests that VD evaluated by OCTA, might be a useful biomarker of disease severity. Our study also showed reduced VD in SRCP in SLE group, but the measurements of FAZ area were opposite to Debourdeau et al. Despite no correlation of VD with disease severity index in our study, kidney involvement among SLE group was associated with further microvasculature reduction.

In Arnould et al. study, VD in SRCP was associated with the cardiovascular (CV) risk profile [39]. This suggests that retina impairment, measured by OCTA, may reflect increased CV risk thus may predict cardiac events. We have not collected data about past CV events nor coexisting CV risk factors e.g., smoking, dyslipidemia, obesity, hypertension, or diabetes. Further studies should collect data about patients CV and rheumatological status for more accurate results.

The strengths of the study were: the size of the rheumatological data, the patients division in terms of duration of HCQ therapy, the calculation of cumulative dose of HCQ and disease activity indices. The major limitations were small sample size and cross-sectional study design and lack of cardiovascular risk profile of patients.

## 5. Conclusions

In conclusion, we demonstrated vessel density reduction in a cohort of SLE patients compared to healthy individuals. Among study group kidney involvement was associated with further microvasculature reduction. FAZ area and FAZ circularity index were reduced in patients suffering from SLE, however, in our opinion, they are not as informative as the VD and may vary among individuals.

Performing the eye angiography with OCTA procedure is considered a milestone in ophthalmology. In authors opinion, growing interest, availability, familiarity with OCTA, as well as awareness of the device advantages will be the reason of more common OCTA usage in interdisciplinary conditions, especially autoimmune diseases. Vessel density assessment obtained by OCTA should be considered at the time of SLE diagnosis and included in disease activity index. Knowing that retinal involvement may precede other organs impairment, the ophthalmological assessment is essential in the overall patient evaluation. Further studies are necessary to answer the question how to implement these findings into patient’s care, including management and treatment modification. The multi-organ changes characterizing SLE emphasize the extremely important role of a holistic, interdisciplinary approach to both disease diagnosis, as well as its management and treatment. Early detection of retinal impairment and use OCTA as a screening modality, may decrease overall disease morbidity. Of great importance are novel diagnostic tools at the time of diagnosis and follow-up because of long-lasting character of all the autoimmune diseases.

## Figures and Tables

**Table 1:** Basic characteristics.

	Study Group (*n* = 30)	Control Group (*n* = 31)	*p* Value
Age (years)	46.07 ± 14.09	44.55 ± 14.11	0.69
Gender (male/female)	5/25	4/27	0.69
Visual acuity (logMAR)	0.01 ± 0.02	0.0	0.02
IOP (mmHg)	15.98 ± 2.4	16.04 ± 2.05	0.82
Disease duration (months)	101.8 ± 89.98	N/A	N/A
SLEDAI-2K	5.2 ± 4.39	N/A	N/A
SLICC/ACR DI	1.3 ± 1.2	N/A	N/A
HCQ cumulative dose (g)	391.23 ± 425.52	N/A	N/A
Anti dsDNA [IU/mL]	316.07 ± 263.16	N/A	N/A
ACL IgM (positive)	12	N/A	N/A
ACL IgG (positive)	29	N/A	N/A
anti-β2 GPI IgG [RU/mL]	28	N/A	N/A
anti-β2 GPI IgM [RU/mL]	19	N/A	N/A
LAC [s]	46.75 ± 31.2	N/A	N/A
C3 [g/L]	0.94 ± 0.31	N/A	N/A
C4 (positive)	5	N/A	N/A
ERS [mm/h]	19.97 ± 24.6	N/A	N/A

IOP—intraocular pressure, SLEDAI-2K—Systemic Lupus Erythematosus Disease Activity Index 2000, SLICC/ACR DI—The Systemic Lupus International Collaborating Clinics/American College of Rheumatology Damage index, HCQ—hydroxychloroquine, Anti dsDNA—Anti-double stranded DNA antibodies, ACL—Anti-cardiolipin antibodies, anti-β2 GPI—Anti-β2-glycoprotein I antibodies, C3, C4—complement system, LAC—lupus anticoagulant, ERS—erythrocyte sedimentation rate, N/A—not applicable. Normal ranges: Anti dsDNA: 0–100 IU/mL—negative, >100 IU/mL—positive; ACL IgM: 0–20 U/mL, ACL IgG: 0–20 U/mL, anti-β2 GPI IgG: 0–20 U/mL, anti-β2 GPI IgM: 0–20 U/mL; LAC: 30.5–40.6 s, C3: 0.75–2.0 g/L, C4: 0.1–0.3 g/L, ERS: 1–15 mm/h.

**Table 2:** Rheumatological treatment.

	Positive	Negative
HCQ < 5 years/HCQ > 5 years	17/13	N/A
Kidney involvement	6	24
CNS involvement	3	27
HCQ	30	0
Chloroquine	12	18
Prednisolone	3	27
Azathioprine	11	19
Cyclophosphamide	4	26
Methotrexate	13	17
Leflunomide	1	29
Rituximab	1	29

HCQ—hydroxychloroquine, CNS—central nervous system, N/A—not applicable.

**Table 3:** Comparison of OCTA parameters.

	Study Group (*n* = 30)	Control Group (*n* = 31)	*p* Value
VD SRCP %—fovea	22.16 ± 3.63	20.14 ± 3.76	0.08
VD SRCP—superior	48.68 ± 2.69	49.52 ± 2.01	0.56
VD SRCP—inferior	48.82 ± 2.36	50.08 ± 2.44	0.04
VD SRCP—nasal	45.82 ± 1.83	46.99 ± 1.57	0.02
VD SRCP—temporal	46.7 ± 2.65	47.45 ± 2.5	0.19
VD SRCP—parafovea	47.51 ± 1.43	48.51 ± 1.5	0.02
VD SRCP—whole en face	44.69 ± 1.4	45.36 ± 1.32	0.13
VD DRCP %—fovea	20.88 ± 4.03	18.94 ± 3.89	0.16
VD DRCP—superior	52.27 ± 2.95	52.98 ± 2.42	0.52
VD DRCP—inferior	52.48 ± 2.43	53.59 ± 2.5	0.12
VD DRCP—nasal	50.08 ± 2.18	50.52 ± 1.99	0.37
VD DRCP—temporal	49.73 ± 2.86	50.31 ± 2.81	0.34
VD DRCP—parafovea	51.14 ± 1.7	51.85 ± 1.73	0.1
VD DRCP—whole en face	47.78 ± 1.59	48.2 ± 1.52	0.39
FAZ—area	0.16 ± 0.07	0.2 ± 0.08	0.03
FAZ—perimeter	1.53 ± 0.36	1.67 ± 0.36	0.11
FAZ—circularity index	0.79 ± 0.09	0.94 ± 0.63	0.01

VD—vessel density, SRCP—superficial retinal capillary plexus, DRCP—deep retinal capillary plexus, FAZ—foveal avascular zone.

**Table 4:** Comparison of OCTA parameters among SLE patients based on HCQ treatment duration.

	>5 Years (*n* = 13)	<5 Years (*n* = 17)	*p* Value
VD SRCP %—fovea	21.8 ± 3.43	22.43 ± 3.86	0.56
VD SRCP—superior	49.74 ± 1.62	47.88 ± 3.09	0.02
VD SRCP—inferior	47.85 ± 2.12	49.57 ± 2.32	0.07
VD SRCP—nasal	46.1 ± 2.14	45.61 ± 1.6	0.65
VD SRCP—temporal	46.49 ± 1.58	46.86 ± 3.28	0.43
VD SRCP—parafovea	47.54 ± 1.23	47.48 ± 1.61	0.9
VD SRCP—whole en face	44.68 ± 1.32	44.69 ± 1.5	0.97
VD DRCP %—fovea	20.63 ± 3.47	21.06 ± 4.51	0.93
VD DRCP—superior	53.3 ± 1.73	51.49 ± 3.47	0.07
VD DRCP—inferior	51.61 ± 2.47	53.15 ± 2.26	0.12
VD DRCP—nasal	50.52 ± 2.34	49.75 ± 2.05	0.45
VD DRCP—temporal	49.62 ± 1.76	49.81 ± 3.53	0.48
VD DRCP—parafovea	51.26 ± 1.32	51.05 ± 1.98	0.97
VD DRCP—whole en face	47.86 ± 1.35	47.72 ± 1.8	0.9
FAZ—area	0.15 ± 0.08	0.16 ± 0.06	0.65
FAZ—perimeter	1.5 ± 0.43	1.55 ± 0.31	0.5
FAZ—circularity index	0.79 ± 0.07	0.78 ± 0.11	0.77

VD—vessel density, SRCP—superficial retinal capillary plexus, DRCP—deep retinal capillary plexus, FAZ—foveal avascular zone.

**Table 5:** Comparison of OCTA parameters among SLE patient using Methotrexate.

	Positive (*n* = 13)	Negative (*n* = 17)	*p* Value
VD SRCP %—fovea	21.33 ± 3	22.79 ± 4.03	0.34
VD SRCP—superior	49.29 ± 1.71	48.22 ± 3.22	0.43
VD SRCP—inferior	47.66 ± 2.24	49.71 ± 2.1	0.03
VD SRCP—nasal	45.99 ± 1.96	45.69 ± 1.78	0.9
VD SRCP—temporal	46.06 ± 1.48	47.19 ± 3.24	0.07
VD SRCP—parafovea	47.25 ± 1.29	47.7 ± 1.54	0.32
VD SRCP—whole en face	44.37 ± 1.18	44.93 ± 1.54	0.21
VD DRCP %—fovea	19.98 ± 3.53	21.56 ± 4.36	0.34
VD DRCP—superior	52.74 ± 1.77	51.92 ± 3.62	0.93
VD DRCP—inferior	51.5 ± 2.51	53.23 ± 2.16	0.05
VD DRCP—nasal	49.81 ± 1.68	50.29 ± 2.52	0.43
VD DRCP—temporal	49.38 ± 1.68	49.99 ± 3.54	0.19
VD DRCP—parafovea	50.86 ± 1.32	51.36 ± 1.96	0.3
VD DRCP—whole en face	47.43 ± 1.21	48.05 ± 1.82	0.18
FAZ—area	0.17 ± 0.07	0.15 ± 0.07	0.28
FAZ—perimeter	1.63 ± 0.34	1.46 ± 0.37	0.22
FAZ—circularity index	0.8 ± 0.07	0.78 ± 0.11	0.62

VD—vessel density, SRCP—superficial retinal capillary plexus, DRCP—deep retinal capillary plexus, FAZ—foveal avascular zone.

**Table 6:** Comparison of OCTA parameters among SLE patient using Azathioprine.

	Positive (*n* = 11)	Negative (*n* = 19)	*p* Value
VD SRCP %—fovea	23.44 ± 4.24	21.42 ± 3.11	0.16
VD SRCP—superior	48.02 ± 3.6	49.07 ± 2.01	0.23
VD SRCP—inferior	49.79 ± 2.39	48.26 ± 2.22	0.14
VD SRCP—nasal	45.22 ± 1.75	46.17 ± 1.83	0.26
VD SRCP—temporal	46.52 ± 3.91	46.8 ± 1.65	1
VD SRCP—parafovea	47.39 ± 1.72	47.57 ± 1.28	0.86
VD SRCP—whole en face	44.73 ± 1.74	44.67 ± 1.21	0.73
VD DRCP %—fovea	22.34 ± 4.95	20.03 ± 3.25	0.23
VD DRCP—superior	51.38 ± 4.06	52.79 ± 2.03	0.25
VD DRCP—inferior	53.11 ± 1.93	52.12 ± 2.66	0.41
VD DRCP—nasal	49 ± 2.13	50.71 ± 1.99	0.05
VD DRCP—temporal	49.02 ± 4.07	50.14 ± 1.87	0.55
VD DRCP—parafovea	50.63 ± 2.01	51.44 ± 1.48	0.21
VD DRCP—whole en face	47.48 ± 1.96	47.95 ± 1.37	0.37
FAZ—area	0.12 ± 0.07	0.18 ± 0.06	0.07
FAZ—perimeter	1.33 ± 0.39	1.65 ± 0.29	0.05
FAZ—circularity index	0.76 ± 0.14	0.8 ± 0.05	0.73

VD—vessel density, SRCP—superficial retinal capillary plexus, DRCP—deep retinal capillary plexus, FAZ—foveal avascular zone.

**Table 7:** Comparison of OCTA parameters among SLE patient using Chloroquine.

	Positive (*n* = 12)	Negative (*n* = 18)	*p* Value
VD SRCP %—fovea	21.14 ± 3.19	22.83 ± 3.84	0.28
VD SRCP—superior	48.81 ± 3.6	48.6 ± 1.99	0.47
VD SRCP—inferior	48.63 ± 3.16	48.95 ± 1.73	0.75
VD SRCP—nasal	45.7 ± 2.22	45.9 ± 1.59	0.48
VD SRCP—temporal	45.6 ± 3.09	47.44 ± 2.08	0.09
VD SRCP—parafovea	47.18 ± 1.88	47.72 ± 1.03	0.39
VD SRCP—whole en face	44.29 ± 1.8	44.96 ± 1.04	0.46
VD DRCP %—fovea	20.02 ± 3.75	21.45 ± 4.22	0.43
VD DRCP—superior	52.11 ± 4	52.38 ± 2.12	0.85
VD DRCP—inferior	51.99 ± 3.06	52.81 ± 1.94	0.41
VD DRCP—nasal	49.68 ± 2.08	50.35 ± 2.26	0.45
VD DRCP—temporal	48.35 ± 3.44	50.64 ± 2.02	0.03
VD DRCP—parafovea	50.54 ± 2.14	51.55 ± 1.25	0.18
VD DRCP—whole en face	47.14 ± 2.03	48.2 ± 1.09	0.17
FAZ—area	0.17 ± 0.06	0.15 ± 0.07	0.69
FAZ—perimeter	1.58 ± 0.29	1.5 ± 0.4	0.78
FAZ—circularity index	0.81 ± 0.06	0.77 ± 0.11	0.11

VD—vessel density, SRCP—superficial retinal capillary plexus, DRCP—deep retinal capillary plexus, FAZ—foveal avascular zone.

**Table 8:** Comparison of OCTA parameters among SLE patient with kidney involvement.

	Positive (*n* = 6)	Negative (*n* = 24)	*p* Value
VD SRCP %—fovea	19.99 ± 2.76	22.7 ± 3.67	0.13
VD SRCP—superior	46.83 ± 3.76	49.15 ± 2.22	0.05
VD SRCP—inferior	49.26 ± 3.21	48.71 ± 2.18	0.82
VD SRCP—nasal	44.26 ± 1.61	46.21 ± 1.7	0.02
VD SRCP—temporal	44.23 ± 3.46	47.32 ± 2.05	0.02
VD SRCP—parafovea	46.14 ± 1.19	47.85 ± 1.29	0.01
VD SRCP—whole en face	43.24 ± 1.29	45.05 ± 1.19	<0.01
VD DRCP %—fovea	18.49 ± 2.02	21.47 ± 4.22	0.11
VD DRCP—superior	50.74 ± 4.73	52.66 ± 2.32	0.36
VD DRCP—inferior	52.96 ± 2.52	52.36 ± 2.45	0.98
VD DRCP—nasal	49.5 ± 2.96	50.23 ± 1.99	0.45
VD DRCP—temporal	47.56 ± 4.49	50.27 ± 2.1	0.14
VD DRCP—parafovea	50.19 ± 2.26	51.38 ± 1.5	0.22
VD DRCP—whole en face	46.67 ± 2.15	48.06 ± 1.34	0.13
FAZ—area	0.19 ± 0.03	0.15 ± 0.07	0.14
FAZ—perimeter	1.72 ± 0.17	1.48 ± 0.38	0.07
FAZ—circularity index	0.81 ± 0.03	0.78 ± 0.1	0.82

VD—vessel density, SRCP—superficial retinal capillary plexus, DRCP—deep retinal capillary plexus, FAZ—foveal avascular zone.

## Data Availability

Not applicable.

## Supplementary Materials

The following supporting information can be downloaded at: https://www.mdpi.com/article/10.3390/jcm11247417/s1, Table S1: Multiple regression analysis between VD SRCP nasal and the following variables; Table S2: Multiple regression analysis between VD SRCP parafovea and the following variables; Table S3: Multiple regression analysis between VD SRCP inferior and the following variables; Table S4: Multiple regression analysis between FAZ area and the following variables; Table S5: Multiple regression analysis between FAZ Circularity index and the following variables; Table S6: Power analysis for comparison of OCTA parameters between SLE and control group; Table S7: Power analysis for multiple regression analysis.
