# Supplementary material for: Evaluation of Subclinical Retinal Disease in Patients Affected by Systemic Lupus Erythematosus with No Evidence of Ocular Involvement—An Optical Coherence Tomography Angiography Original Study
Source: J Clin Med. 2022 Dec 14;11(24):7417. doi: 10.3390/jcm11247417 (PMC9780932; doi:10.3390/jcm11247417)
Supplement: Supplementary file 1 [file jcm-11-07417-s001.zip › jcm-2057061-supplementary.pdf]

Table S1. Multiple regression analysis between VD SRCP nasal and the following variables. R = 0.64617387

	b	t(19)	P value
Age	0.01094	0.41584	0.68
Sex	-0.81880	-0.75506	0.46
SLEDAI-2K	-0.07936	-0.95577	0.35
Disease duration	-0.00709	-1.41446	0.17
SLICC/ACR Damage Index	0.37849	1.32436	0.20
Kidney involvement	-1.89780	-2.06566	0.05
HCQ cumulative dose	0.00115	0.93377	0.36
Azathioprine	-0.74789	-0.90726	0.38
Methotrexate	0.23124	0.30574	0.76
HCQ duration	-0.61076	-0.52763	0.60

Durbin-Watson d value - 1,345189.

Table S2. Multiple regression analysis between VD SRCP parafovea and the following variables. R= 0.63569661

	b	t(19)	P value
Age	-0.02895	-1.39298	0.18
Sex	-0.63079	-0.73669	0.47
SLEDAI-2K	0.00300	0.04582	0.96
Disease duration	-0.00125	-0.31602	0.76
SLICC/ACR Damage Index	0.17524	0.77659	0.45
Kidney involvement	-2.00618	-2.76551	0.01
HCQ cumulative dose	0.00061	0.62332	0.54
Azathioprine	-0.20132	-0.30930	0.76
Methotrexate	-0.39426	-0.66018	0.52
HCQ duration	-0.49641	-0.54311	0.59

Durbin-Watson d value - 1,848533.

Table S3. Multiple regression analysis between VD SRCP inferior and the following variables. R= 0.58803251

	b	t(19)	P value
Age	0.01204	0.33477	0.74
Sex	-1.62369	-1.09577	0.29
SLEDAI-2K	-0.09192	-0.81012	0.43
Disease duration	-0.00217	-0.31616	0.76
SLICC/ACR Damage Index	0.02693	0.06895	0.95
Kidney involvement	-0.05766	-0.04593	0.96
HCQ cumulative dose	-0.00013	-0.07758	0.94
Azathioprine	1.43384	1.27294	0.22
Methotrexate	-1.70418	-1.64900	0.12
HCQ duration	-0.97257	-0.61488	0.55

Durbin-Watson d value - 2,049834.

Table S4 Multiple regression analysis between FAZ area and the following variables. R= 0.61252037

	b	t(19)	P value
Age	0.000791	0.79146	0.44
Sex	-0.007715	-0.18747	0.85
SLEDAI-2K	-0.000333	-0.10556	0.92
Disease duration	-0.000041	-0.21345	0.83
SLICC/ACR Damage Index	0.006843	0.63098	0.54
Kidney involvement	0.025925	0.74357	0.47
HCQ cumulative dose	-0.000044	-0.93633	0.36
Azathioprine	-0.065260	-2.08605	0.05
Methotrexate	0.023060	0.80341	0.43
HCQ duration	-0.008870	-0.20192	0.84

Durbin-Watson d value - 2,124973.

Table S5 Multiple regression analysis between FAZ Circularity index and the following variables. R= 0.48202293

	b	t(19)	P value
Age	0.000374	0.24391	0.81
Sex	0.096357	1.52502	0.14
SLEDAI-2K	0.000814	0.16822	0.87
Disease duration	0.000129	0.44291	0.66
SLICC/ACR Damage Index	-0.022036	-1.32338	0.20
Kidney involvement	0.054582	1.01964	0.32
HCQ cumulative dose	0.000017	0.24157	0.81
Azathioprine	-0.061406	-1.27848	0.22
Methotrexate	0.023240	0.52737	0.60
HCQ duration	-0.007949	-0.11786	0.91

Durbin-Watson d value - 2,280719.

Table S6 Power analysis for comparison of OCTA parameters between SLE and control group.

Analyzed parameter	Mean +/- SD SLE	Mean +/- SD Control	Alfa	Power value	Required sample size
VD SRCP % - fovea	22.16 ± 3.63	20.14 ± 3.76	0,05	0,8	52
VD SRCP - superior	48.68 ± 2.69	49.52 ± 2.01	0,05	0,8	162
VD SRCP - inferior	48.82 ± 2.36	50.08 ± 2.44	0,05	0,8	57
VD SRCP - nasal	45.82 ± 1.83	46.99 ± 1.57	0,05	0,8	40
VD SRCP - temporal	46.7 ± 2.65	47.45 ± 2.5	0,05	0,8	197
VD SRCP - parafovea	47.51 ± 1.43	48.51 ± 1.5	0,05	0,8	34
VD SRCP - whole en face	44.69 ± 1.4	45.36 ± 1.32	0,05	0,8	70
VD DRCP % - fovea	20.88 ± 4.03	18.94 ± 3.89	0,05	0,8	69
VD DRCP - superior	52.27 ± 2.95	52.98 ± 2.42	0,05	0,8	272
VD DRCP - inferior	52.48 ± 2.43	53.59 ± 2.5	0,05	0,8	77
VD DRCP - nasal	50.08 ± 2.18	50.52 ± 1.99	0,05	0,8	387
VD DRCP - temporal	49.73 ± 2.86	50.31 ± 2.81	0,05	0,8	383
VD DRCP - parafovea	51.14 ± 1.7	51.85 ± 1.73	0,05	0,8	91
VD DRCP - whole en face	47.78 ± 1.59	48.2 ± 1.52	0,05	0,8	226
FAZ - area	0.16 ± 0.07	0.2 ± 0.08	0,05	0,8	50
FAZ - perimeter	1.53 ± 0.36	1.67 ± 0.36	0,05	0,8	105
FAZ - circularity index	0.79 ± 0.09	0.94 ± 0.63	0,05	0,8	7

Table S7 Power analysis for multiple regression analysis

Analyzed parameter	P^2	Alfa	Power value	Required sample size
VD SRCP nasal	0,41	0,05	0,9	41
VD SRCP parafovea	0,09	0,05	0,9	213
VD SRCP inferior	0,35	0,05	0,9	50
FAZ area	0,37	0,05	0,9	47
FAZ circularity index	0,23	0,05	0,9	79
